# In Vivo Imaging of Inflammation and Infection 2019

**DOI:** 10.1155/2020/6824583

**Published:** 2020-02-11

**Authors:** Anne Roivainen, Xiang-Guo Li, Sarah Ohrndorf, Conny J. van der Laken

**Affiliations:** ^1^Turku PET Centre, University of Turku, FI-20521 Turku, Finland; ^2^Turku PET Centre, Turku University Hospital, FI-20521 Turku, Finland; ^3^Turku Center for Disease Modeling, University of Turku, FI-20521 Turku, Finland; ^4^Turku PET Centre, Åbo Akademi University, FI-20521 Turku, Finland; ^5^Charité – Universitätsmedizin, Berlin, Germany; ^6^Department of Rheumatology, Amsterdam University Medical Center Location VU University Medical Center, Amsterdam, Netherlands

Inflammation is involved in a number of medical conditions, and imaging-based diagnosis is frequently used for timely identification and localization of inflammatory foci. Inflammation is closely related to infection, and it is necessary to take inflammation into account in the imaging process of infection. Conventional imaging techniques (computed tomography, magnetic resonance imaging, and ultrasound) may detect such pathology but mainly rely on anatomical changes and are not fully capable of discriminating active inflammatory pathology from the anatomical changes resulting from prior successful therapy or surgery. In addition, inflammatory foci cannot be detected in the early phase of development because of the lack of substantial anatomical changes at this time. Nuclear imaging with radiotracers that accumulate at the site of inflammation has become an established tool in the evaluation of several inflammatory and infectious conditions. They can reveal molecular and cellular changes and provide sensitive detection of even small inflammatory and/or infectious foci at an early stage of disease.

This special issue consists of totally seven articles (72 pages) including three review articles, three research articles, and one clinical study. Although most of the articles are dealing with positron emission tomography/computed tomography (PET/CT) (four articles), magnetic resonance imaging (MRI, two articles) and ultrasound, as well as optical imaging techniques are discussed. The contents of this special issue cover from early phases of ligand design, chemistry development, and preclinical evaluations to clinical imaging protocols and techniques.

Because during inflammation glucose consumption is raised due to active inflammatory cells, such as macrophages, and rapidly proliferating healing tissues, glucose analog 2-deoxy-2-[^18^F]fluoroglucose (^18^F-FDG) facilitates PET imaging of inflammation. ^18^F-FDG is very sensitive and useful to detect both acute and chronic inflammation although it is not an inflammation-specific agent. The clinical study by K. Taimen et al. “The Clinical Impact of Using ^18^F-FDG-PET/CT in the Diagnosis of Suspected Vasculitis: The Effect of Dose and Timing of Glucocorticoid Treatment” pointed at a clinically relevant issue of the impact of timing and dose of glucocorticoids on the diagnostic value of ^18^F-FDG-PET/CT for large vessel vasculitis. They demonstrated that ^18^F-FDG-PET/CT positivity of large vessels was significantly associated with a lower dose and shorter duration of glucocorticoid medication and higher CRP level in vasculitis patients. The review article by G. Treglia “Diagnostic Performance of ^18^F-FDG PET/CT in Infectious and Inflammatory Diseases according to Published Meta-Analyses” presents on the basis of the current evidence—taking 36 meta-analyses into account—a good diagnostic performance of ^18^F-FDG PET/CT for several inflammatory and infectious diseases, in particular, cardiovascular infectious and inflammatory diseases and some musculoskeletal infections. In future, more prospective multicenter studies and cost-effective analyses are warranted. Although ^67^Ga-citrate has been used for decades in single-photon emission computed tomography (SPECT) imaging of inflammation, it has largely been replaced by ^18^F-FDG PET. However, positron-emitting analog ^68^Ga-citrate may also be useful in certain indications. The research article by Z. Wang et al. “Comparative Evaluation of ^68^Ga-Citrate PET/CT and ^18^F-FDG PET/CT in the Diagnosis of Type II Collagen-Induced Arthritis in Rats” suggests that ^68^Ga-citrate PET/CT may be helpful for detecting inflammatory activity of affected joints, e.g., in patients with rheumatoid arthritis.

It is very important to distinguish between infection and sterile inflammation although there are always inflammatory components also in the infection. Novel imaging agents are being developed in order to distinguish infection and inflammation. The research article by T. A. Aweda et al. “Radiolabeled Cationic Peptides for Targeted Imaging of Infection” has described a bacterial membrane targeting strategy with peptide-based imaging ligands. For example, in the structure of one of the ligands, an unnatural amino acid AB1 has been used to form covalent bonds with the amino group containing bacterial membrane lipids.

The review article by N. Jugniot et al. “Neutrophil Elastase Activity Imaging: Recent Approaches in the Design and Applications of Activity-Based Probes and Substrate-Based Probes” describes the recent progress of ligand development for imaging neutrophil elastase activity. The protease neutrophil elastase has a role in diseases including lung inflammation. The imaging probes are designed based on the corresponding mechanisms of enzyme-ligand interactions for imaging with different modalities.

The article by U. Jehn et al. “Renal Allograft Rejection: Noninvasive Ultrasound- and MRI-Based Diagnostics” reviews the current state-of-the-art approaches for noninvasive diagnostics of acute renal transplant inflammation, i.e., rejection, especially focusing on nonradiation-based methods using MRI and ultrasound. Another MRI-related paper, the research article by D. Ippolito et al. “Dynamic Contrast-Enhanced MR with Quantitative Perfusion Analysis of Small Bowel in Vascular Assessment between Inflammatory and Fibrotic Lesions in Crohnʼs Disease: A Feasibility Study” suggests that functional MRI with quantitative and semiquantitative measurements may represent a complementary diagnostic tool for evaluation of local inflammation activity in patients with Crohn's disease.

This special issue presents novel imaging probes, preclinical imaging evidences, and clinical applications of imaging-based diagnosis in inflammatory and infectious conditions, in addition to comprehensive, systematic, but concise overviews in this field. We hope that this issue will serve as a small forum in this specific field, and further research will be carried out in the research community.

